# TOETVA parathyroid autofluorescence detection: hANDY-i endoscopy attachment

**DOI:** 10.3389/fendo.2023.1233956

**Published:** 2023-08-24

**Authors:** Stefanie Seo, Khalid Mohamed Ali, Samantha A. Wolfe, Nimesh V. Nagururu, Andy S. Ding, Dipan Desai, R. Alex Harbison, Yoseph Kim, Bo Ning, Richard Jaepyeong Cha, Jonathon O. Russell

**Affiliations:** ^1^ Department of Otolaryngology – Head and Neck Surgery, Johns Hopkins School of Medicine, Baltimore, MD, United States; ^2^ Department of Endocrine Surgery, University Health Network, Toronto, ON, Canada; ^3^ Department of Research, Optosurgical, LLC, Columbia, MD, United States; ^4^ Sheikh Zayed Institute, Children’s National Hospital, Washington, DC, United States; ^5^ Department of Pediatrics, George Washington University School of Medicine and Health Sciences, Washington, DC, United States

**Keywords:** parathyroid glands, near-infrared autofluorescence, endoscopy, remote-access thyroidectomy, thyroidectomy

## Abstract

**Background:**

Treatment options for thyroid pathologies have expanded to include scarless and remote access methods such as the transoral endoscopic thyroidectomy vestibular approach (TOETVA). Currently, no standardized methods exist for locating parathyroid glands (PGs) in patients undergoing TOETVA, which can lead to parathyroid injury and subsequent hypocalcemia. This early feasibility study describes and evaluates the hANDY-i endoscopic attachment for detecting PGs in transoral thyroidectomy.

**Methods:**

We used a prototype parathyroid autofluorescence imager (hANDY-i) that was mounted to a 10-mm 0-degree endoscope. The device delivers a split screen view of Red-green-blue (RGB) and near-infrared autofluorescence (NIRAF) which allows for simultaneous anatomical localization and fluorescence visualization of PGs during endoscopic thyroid dissection.

**Results:**

One cadaveric case and two patient cases were included in this study. The endoscopic hANDY-i imaging system successfully visualized PGs during all procedures.

**Conclusion:**

The ability to leverage parathyroid autofluorescence during TOETVA may lead to improved PG localization and preservation. Further human studies are needed to assess its effect on postoperative hypocalcemia and hypoparathyroidism.

## Introduction

Accurate intraoperative identification of parathyroid glands (PG) is critical for gland preservation and prevention of hypoparathyroidism in transoral endoscopic thyroidectomy vestibular approach (TOETVA), a recently described procedure that has gained global popularity for thyroid and parathyroid procedures (1). TOETVA owes its attractiveness in particular to its avoidance of virtually all cutaneous scars (2). Compared to the standard transcervical thyroidectomy approach (TCA), TOETVA is predicted to decrease psychological morbidity associated with cervical scars and increase overall quality of life (3–5). While complication rates in this newer technique are comparable to those in TCA, permanent or transient hypoparathyroidism remains the most commonly reported complication (6).

Currently, no standardized methods exist for locating PGs in patients undergoing thyroidectomies. Indocyanine green (ICG) was previously a widely used exogenous dye to help improve PG visualization, but its setbacks were allergic reactions to dye and slow onset of fluorescence signals (7). Near-infrared autofluorescence (NIRAF) is a novel technique that utilizes the intrinsic biological fluorophores of PG cells for noninvasive and automated identification (8). While there are FDA-approved NIRAF devices, Fluobeam LX and PTeye, these systems are not compatible with the laparoscopic surgical approach to the central neck. Our team has recently prototyped and reported feasibility of a handheld optical module called hANDY-i in open thyroidectomy procedures (9). The hANDY-i endoscopic attachment is an adaptation of our device for transoral procedures and allows identification of PGs without the need for any additional incisions. In the landscape of many newly emerging PG detection devices, this paper describes one of the earliest transoral applications of a PG imaging system.

## Materials and methods

**Figure 1:**
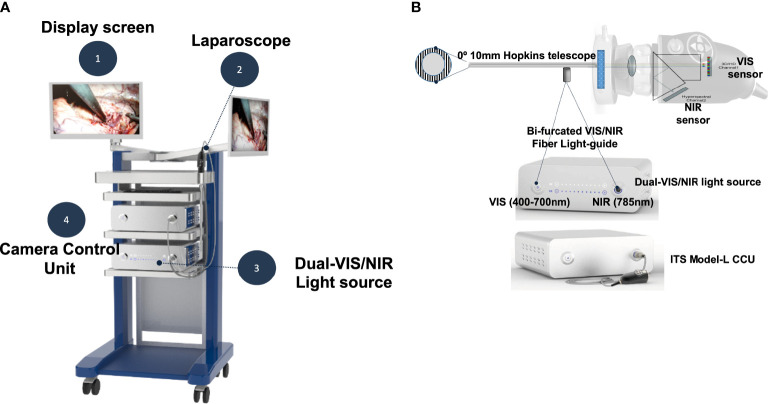
The TOETVA autofluorescence imaging system consists of four parts as shown **(A)**. The camera head (ITS Model-L) includes both visible and near-infrared (NIR) sensors **(B)**. The NIR sensor detects autofluorescence emission light (>820nm) from parathyroid glands. A custom-designed bifurcated fiber light-guide was used to transmit both visible and near-infrared laser light into a 0-degree Hopkins telescope.

hANDY-i is one of the earliest reported non-invasive, handheld devices that produces a combined RGB and NIRAF view of PGs during thyroid or parathyroid surgery (9). This prototype employs two imaging modalities to reveal PGs in a superimposable, split screen view within the surgical workflow and can be used for both open and transoral cases. hANDY-i consists of a coaxial 785 nm fiber laser, dual-sensors for RGB and NIRAF imaging, and an embedded processing board (**Figure 1A**). This camera module was connected to a 10mm 0-degree endoscope that allows for use during transoral surgeries (**Figure 1B**). The connected display monitor shows two superimposable images: a true-color view of the operating field in mode 1 and label-free NIRAF localization of PGs in mode 2.

**Figure 2:**
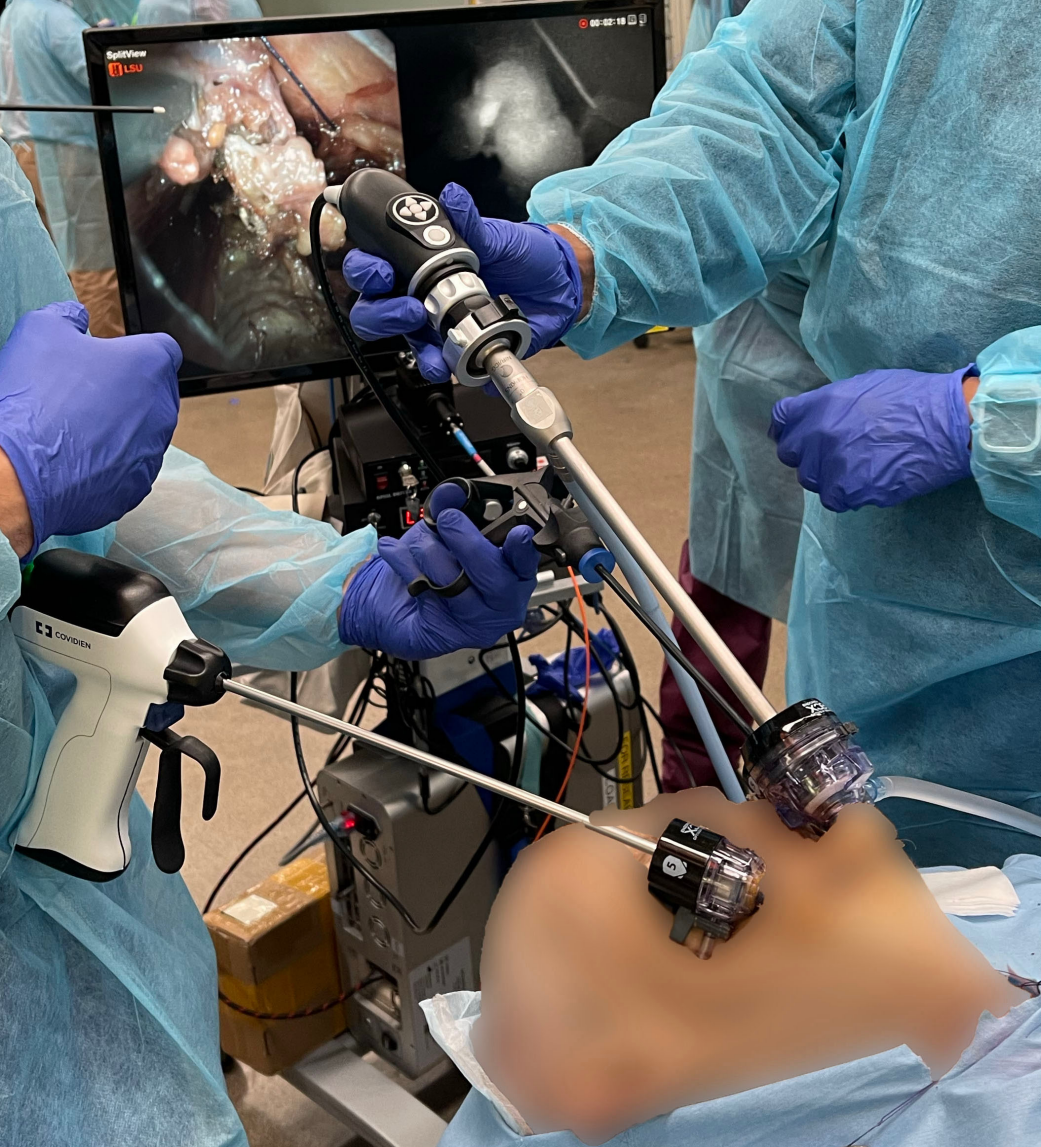
Standard set-up of hANDY-i for TOETVA thyroidectomy: the camera endoscope was inserted through the 10mm central trocar.

Our early feasibility study consisted of one cadaveric case and two patient cases. This study was approved by the Johns Hopkins Institutional Review Board (IRB00224302). The operating surgeon followed the standard TOETVA procedure consisting of a surgical incision to the oral vestibule, expansion of the vestibular access, use of blunt dissectors to expand the subplatysmal space with enlargement of the surgical field, and placement of trocars. The hANDY-i endoscopic attachment was inserted through the 10mm central trocar (**Figure 2**). Mode 1 displayed the RGB color view of the operating field. Mode 2 was used to isolate PGs, seen as superimposable, hyperfluorescent spots on a hypofluorescent background. PG location was confirmed by visual inspection. The visual cues used by the surgeon were (1) yellow-brown color, (2) typical ovoid shape, and (3) distinct vasculature. Parathyroid vascularization was qualitatively evaluated by assessing the color of the gland and visualizing evidence of bleeding from the microcirculation following removal of the thyroid.

## Results

### Case 1: cadaver

**Figure 3:**
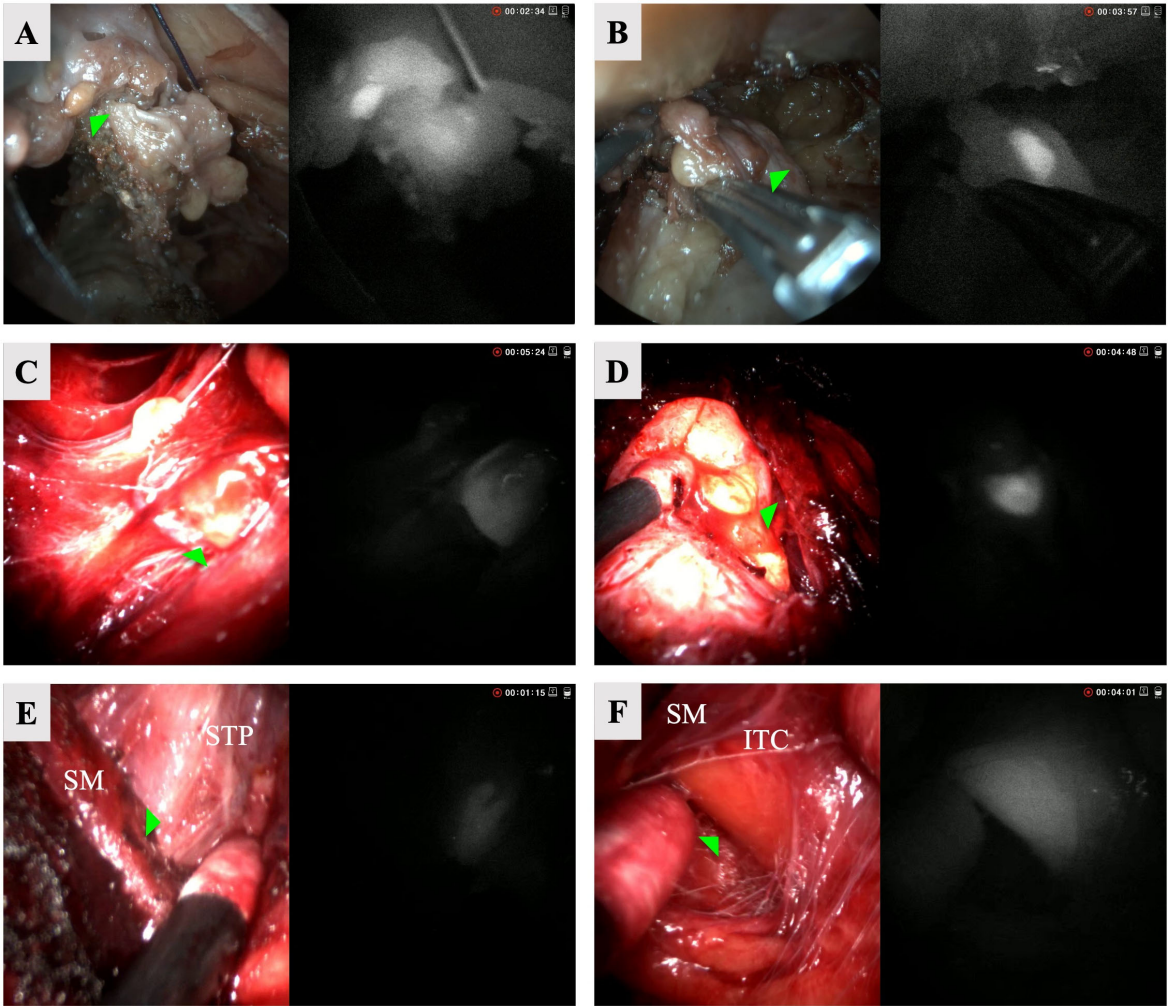
RGB (left) and NIRAF (right) split screen views of six parathyroid glands accessed via TOETVA. Row 1: left superior **(A)** and left subscapular **(B)** from cadaver. Row 2: left superior **(C)** and right inferior **(D)** from patient 1. Row 3: left superior **(E)** and left inferior (intracapsular) **(F)** parathyroid glands from patient 2. STP, Superior thyroid pedicle; SM, strap muscles; ITC, inferior thyroid capsule.

The left superior (**Figure 3A**) and a left subcapsular, intrathyroidal (**Figure 3B**) glands were detected with hANDY-i. The second gland was difficult to visualize by the surgeon’s naked eye, but hANDY-i showed an area of fluorescence along the inferolateral edge of the thyroid lobe corresponding to a subcapsular, intrathyroidal PG. Next, the entire thyroid lobe specimen was removed and re-examined ex-vivo with hANDY-i. The fluorescent tissues were dissected from the surrounding thyroid parenchyma and inspected with Fluobeam LX, which confirmed positive fluorescent results.

### Case 2: patient 1

The patient was a 38-year-old female with Graves’ disease with nodular evolution who underwent a total thyroidectomy for a 7 cm left benign nodule and a 2 cm right benign nodule. The left superior (**Figure 3C**), right superior, and right inferior (**Figure 3D**) glands were detected with hANDY-i and preserved *in situ*. Re-inserting the endoscope, it was noted that the left superior and right inferior glands appeared devascularized upon visual inspection. They were subsequently removed from the patient, minced, and autotransplanted into the respective sternohyoid muscles. Post-operative PTH and calcium were 9 pg/mL and 9.3 mg/dL respectively, and the patient was started on 30 days of calcium supplementation. At one month follow-up, her PTH corrected to 27 pg/mL.

### Case 3: patient 2

The patient was a 47-year-old female who underwent a left lobectomy for a 4 cm benign thyroid nodule. The left superior (**Figure 3E**) and left inferior glands were detected with hANDY-i. Initially, a separate PG-like tissue on the laterally reflected portion was thought to be the left inferior gland, but hANDY-i did not show autofluorescence (AF) in this tissue and instead located AF in the capsule (**Figure 3F**), indicating that the true PG was still intracapsular. The inferior PG was meticulously dissected from the thyroid gland and anatomically preserved maintaining the tissue encompassing the pedicle of the inferior thyroid artery. The parathyroid gland was therefore excluded from the final thyroid specimen. Maintaining anatomic orientation, both glands were well-perfused upon visual inspection based on the color and presence of blood flow and left *in situ*. Calcium and PTH values were not drawn per standard practice for thyroid lobectomies at our institution.

## Discussion

Recent developments in remote access thyroidectomy techniques have necessitated imaging modalities that meet new operative needs. Currently there are two FDA approved NIRAF parathyroid detection devices: Fluobeam LX and PTeye. While Fluobeam has a sensitivity and specificity of 94.1% and 80% respectively, the device’s high light sensitivity requires a dark operating field, potentially hindering its incorporation into the workflow (10). PTeye is a similar device with the distinction of a probe-based design. PTeye has a comparable overall accuracy of 94.3%, but the lack of a video feedback and need for patient-specific calibration complicate its use in the operating room (11). While Fluobeam LX and PTeye can only be utilized in open transcervical thyroidectomy, there have been recent developments in endoscopic PG detection devices. The da Vinci Firefly and EleVision are the first two reported devices designed to be compatible with endoscopic thyroidectomy. The da Vinci Firefly is an ICG fluorescence-based technology that was first introduced in 2017. While the authors showed its applicability in bilateral axillo-breast approach (BABA) robotic thyroidectomy, Firefly is not PG specific and utilizes ICG fluorescence instead of NIRAF (12). EleVision bears more similarities to hANDY-i in that it displays NIRAF and ICG angiography and can be mounted to an endoscopic device (13). EleVision successfully located PGs in the first three transoral thyroidectomy cases as described in a 2022 communication (14). To date, the endoscopic applicability of Firefly and EleVision, as with hANDY-i, is still in early testing phases with promising results but few reported cases available in literature.

In this feasibility study, the endoscopic attachment of hANDY-i was used to achieve detection of PGs in both cadaveric and clinical scenarios of transoral thyroid surgery. In all seven of the PGs identified, the hANDY-i fluorescence signal correctly corresponded to the true PG confirmed by the surgeon’s visual inspection. Of these, two PGs were initially discovered only by AF, then subsequently confirmed to be PGs with Fluobeam LX AF and surgeon inspection. This outcome highlights a strength of NIR technologies in detecting even PGs that are buried in other tissue or lack standard visual cues. However, as this is an early feasibility report, future studies with larger case series and randomized controlled trials will be necessary to clearly show the advantage of hANDY-i for reducing post-operative hypoparathyroidism.

A limitation of any image-based identification system is that only PGs that are within the visual field can be identified. For example, in the cadaveric case, the left inferior PG was initially not visualized on endoscopy and subsequently excised with the thyroid specimen. In normal surgical scenarios, this PG would have been devitalized. A second limitation is that PG localization with this method does not inform the surgeon of its viability, which limits its clinical utility in prognosticating the risk of postoperative hypocalcemia. Further improvements in technology are needed to overcome both limitations.

Advancements in video-assisted endoscopic surgery hold a wealth of opportunities for patient outcome improvement beyond the capacity of open surgeries. Remote access thyroidectomy methods have significantly improved cosmetic outcomes (15, 16) while achieving comparable complication rates (17–22), garnering increasing attention globally since the first completely video-assisted endoscopic thyroid lobectomy was introduced in 1997 (23–25). Additional features that allow assessment of PG viability in real time without the need for ICG or other injectables will add value to patients and surgeons. Moreover, the ability to display both NIR and tissue viability within an endoscopic system would allow for rapid transition between technologies that promises improved imaging and clinical decision making. Pairing these technologies in a single system that does not disrupt normal operative workflow may lead to improved patient safety and decreased costs in a manner that improves adoption.

## Conclusion

In conclusion, the endoscopic prototype of hANDY-i extends the applicability of our device to transoral thyroid surgery, a minimally invasive technique that continues to rise in demand. This is one of the first early feasibility studies on a TOETVA-specific PG detection system. Notably, hANDY-i achieved identification of two nontraditional PGs which were initially missed during standard visual inspection. Larger scale clinical applications are necessary to corroborate these findings.

## Author’s note

The content is solely the responsibility of the authors and does not necessarily represent the official views of the National Institutes of Health.

## Data availability statement

The raw data supporting the conclusions of this article will be made available by the authors, without undue reservation.

## Ethics statement

The studies involving human participants were reviewed and approved by Johns Hopkins Institutional Review Board (IRB00224302). The patients/participants provided their written informed consent to participate in this study. Written informed consent was obtained from the individual(s) for the publication of any potentially identifiable images or data included in this article.

## Author contributions

SS: Conception/design, data acquisition, data analysis, manuscript drafting, data presentation, final approval. KA: Conception/design, data acquisition, manuscript drafting, final approval. SW: Conception/design, data acquisition, manuscript drafting, final approval. NN: Conception/design, data acquisition, manuscript drafting, final approval. AD: Conception/design, data acquisition, manuscript drafting, final approval. DD: Conception/design, data acquisition, manuscript drafting, final approval. AH: Conception/design, data acquisition, manuscript drafting, final approval. YK: Conception/design, data acquisition, manuscript drafting, final approval. BN: Conception/design, data acquisition, manuscript drafting, final approval. RC: Conception/design, data acquisition, manuscript drafting, final approval. JR: Conception/design, data acquisition, manuscript drafting, final approval. All authors contributed to the article and approved the submitted version.
